# *q*-Sumudu Transforms of *q*-Analogues of Bessel Functions

**DOI:** 10.1155/2014/327019

**Published:** 2014-01-29

**Authors:** Faruk Uçar

**Affiliations:** Department of Mathematics, Marmara University, Kadıköy, 34722 Istanbul, Turkey

## Abstract

The main purpose of this paper is to evaluate *q*-Sumudu transforms of a product of *q*-Bessel functions. Interesting special cases of theorems are also discussed. Further, the results proved in this paper may find certain applications of *q*-Sumudu transforms to the solutions of the *q*-integrodifferential equations involving *q*-Bessel functions. The results may help to extend the *q*-theory of orthogonal functions.

## 1. Introduction

The Sumudu transform introduced by Watugala [1] has a close resemblance with the Laplace transform but has a wider frequency domain and is better suited to solve intricate problems in engineering and applied sciences. The main advantage of the Sumudu transform is that it may be used to solve problems without resorting to a new frequency domain, because it preserves scale and unit properties (see [2]). For further details, readers may refer to the recent papers, for example, [3–5], on this subject. It is well known that in the literature there are many *q*-extensions of the Bessel function rearranged by Ismail [6]. Here we are concerned with *q*-Sumudu transform of *q*-analogues of these *q*-Bessel functions. Purohit and Kalla [7] evaluated the *q*-Laplace transforms of a product of basic analogues of the Bessel functions. They gave several useful special cases as application. Recently, Albayrak et al. [8] have investigated the fundamental properties of the *q*-Sumudu transforms and established several theorems related to *q*-images of some elementary functions. Subsequently, the same authors evaluated the *q*-Sumudu images of a number of *q*-polynomials and *q*-hypergeometric functions (see [9]).

## 2. Definitions and Preliminaries

In this section, we purpose to add one more dimension to this study by giving some theorems which give rise to *q*-Sumudu images of a product of *q*-Bessel functions. *q*-Bessel functions were introduced by Jackson [10] and are therefore referred to as Jackson's *q*-Bessel functions. Some *q*-analogues of the Bessel functions are given by

(1) Jν(1)(z;q)=(qν+1;q)∞(q;q)∞(z2)ν ×Φ21[00qν+1;q,−z24], |z|<2=(z2)ν∑n=0∞(−z2/4)n(q;q)ν+n(q;q)n,

(2) Jν(2)(z;q)=(qν+1;q)∞(q;q)∞(z2)ν ×Φ01[−qν+1;q,−qν+1z24], (z∈ℂ)=(z2)ν∑n=0∞qn(n+ν)(−z2/4)n(q;q)ν+n(q;q)n

(3)     =(qν+1;q)∞(q;q)∞(z2)v ×∑k=0∞qn(n−1)(qv+1;q)n(q;q)n(−qν+1z24)n.

Hahn [11] showed that these *q*-Bessel functions are related by the following equality:

(4) Jν(2)(z;q)=(−z24;q)∞Jν(1)(z;q), |z|<2.

Both these *q*-analogues have been studied extensively by Ismail in [6, 12]. The third kind of *q*-analogue of the Bessel function is given by

(5) Jν(3)(z;q)=(qν+1;q)∞(q;q)∞zν ×Φ11[0qν+1;q,qz2], (z∈ℂ)=zν∑n=0∞(−1)nqn(n−1)/2(qz2)n(q;q)ν+n(q;q)n.

This third kind of  *q*-Bessel function is also defined by Jackson and sometimes it is called Hahn-Exton *q*-Bessel function. The notation _1_Φ_1_ in (5) is the standard in use of   *q*-hypergeometric series [13]. These *q*-analogues of the Bessel function satisfy the following relations [14]:

(6) lim⁡q→1−Jν(k)((1−q)z;q)=Jν(z), (k=1,2),lim⁡q→1−Jν(3)((1−q)z;q)=Jν(2z).

To make this work easy to read, we need some notations and preliminaries about the quantum theory. For any real number *α*, the *q*-analogue of *α* is defined by

(7) $$\begin{array}{c} \left[\alpha \right]∶=\frac{q^{\alpha }-1}{q-1}. \end{array}$$

The following usual notations are very useful in the theory of *q*-calculus:

(8) (a;q)n=∏k=0n−1(1−aqk), for  n=1,2,…,  (a;q)0=1,(a;q)∞=∏k=0∞(1−aqk),  (a;q)t=(a;q)∞(aqt;q)∞ (t∈ℝ).

See [13] for all of the above definitions and the related formulas. Furthermore, *q*-hypergeometric functions are defined by [13]

(9) Φrs[a1a2⋯ar;q,zb1b2⋯bs] =∑n=0∞(a1,a2,…,ar;q)n(b1,b2,…,bs;q)n[(−1)nq(n2)]s+1−rzn(q;q)n,

where the *q*-shifted factorial, for *a*_*i*_ ∈ *ℂ* (*i* = 1,2,…, *r*), is

(10) (a1,a2,…,ar;q)n=∏i=1r(ai;q)n.

Albayrak et al. [8] have defined *q*-analogues of the Sumudu transform by means of the following *q*-integrals:

(11) Sq{f(t);s} =1(1−q)s∫0sEq(qst)f(t)dqt, s∈(−τ1,τ2),

over the set of functions

(12) A={f(t) ∣ ∃M,τ1,τ2>0, |f(t)|<MEq(|t|τj), t∈(−1)j×[0,∞)},

(13) 𝕊q{f(t);s}=1(1−q)s∫0∞eq(−1st)f(t)dqt                 s∈(−τ1,τ2),

over the set of functions

(14) B={f(t) ∣ ∃M,τ1,τ2>0, |f(t)|<Meq(|t|τj), t∈(−1)j×[0,∞)},

where *q*-analogues of the classical exponential functions are defined by

(15) eq(t)=∑n=0∞tn(q;q)n=1(t;q)∞, (|t|<1),Eq(t)=∑n=0∞  (−1)nqn(n−1)/2tn(q;q)n=(t;q)∞, (t∈ℂ).

Jackson [15] introduced the *q*-integrals as follows

(16) ∫0xf(t)dqt=x(1−q)∑k=0∞qkf(xqk),∫0∞/Af(t)dqt=(1−q)∑k∈ℤqkAf(qkA).

By virtue of (16), *q*-Sumudu transforms can be expressed as

(17) $$\begin{array}{c} S_{q}\left\{f\left(t\right);s\right\}={\left(q;q\right)}_{\infty }\sum_{k=0}^{\infty }\frac{q^{k}}{{\left(q;q\right)}_{k}}f\left(sq^{k}\right), \end{array}$$

(18) $$\begin{array}{c} {𝕊}_{q}\left\{f\left(t\right);s\right\}=\frac{s^{-1}}{{\left(-\left(1/s\right);q\right)}_{\infty }}\sum_{k\in \mathbb{Z}}q^{k}f\left(q^{k}\right){\left(-\frac{1}{s};q\right)}_{k}. \end{array}$$

Integral representations of *q*-gamma function are defined by

(19) Γq(α)=∫01/(1−q)xα−1Eq(q(1−q)x)dqx, (α>0),Γq(α)=K(A;α)∫0∞/A(1−q)xα−1eq(−(1−q)x)dqx                         (α>0),

where *K*(*A*; *α*) is the following remarkable function [16, p. 15]:

(20) K(A;α)=Aα−1(−q/α;q)∞(−qt/α;q)∞(−α;q)∞(−αq1−t;q)∞        (α∈ℝ).

The *q*-gamma and *K*(*A*; *α*) function have the following properties [16, p. 15]:

(21) Γq(x)=(q;q)∞(qx;q)∞(1−q)1−x=(q;q)x−1(1−q)x−1,

(22) K(A;α)=qα−1K(A;α−1).

By virtue of (16), *q*-gamma function can be expressed as

(23) Γq(α)=(q;q)∞(1−q)α−1∑k=0∞qkα(q;q)k,

(24) Γq(α)=K(A;α)(1−q)α−1(−(1/A);q)∞∑k∈ℤ(qkA)α(−1A;q)k.

## 3. Main Theorems

In this section we will evaluate *q*-Sumudu transforms of a product of *q*-Bessel functions.

**Theorem 1**

(a) Let $J_{2\mu_{j}}^{\left(1\right)}\left(2\sqrt{a_{j}t};q\right)$, *j* = 1,2,…, *n*, be *n* different *q*-Bessel functions and

(25) $$\begin{array}{c} f\left(t\right)=t^{\upsilon -1}\prod_{j=1}^{n}J_{2\mu_{j}}^{\left(1\right)}\left(2\sqrt{a_{j}t};q\right). \end{array}$$

Then, *q*-Sumudu transform of *f*(*t*) is

(26) Sq{f(t);s} =(1−q)υ−M−1a1μ1⋯anμnΓq(υ+M)sυ+M−1Γq(2μ1+1)⋯Γq(2μn+1)  ×∑m1,…,mn=0∞(qυ+M;q)m  ×∏j=1n(−ajs)mj(q2μj+1;q)mj(q;q)mj,

where *m* = *m*_1_ + *m*_2_ + ⋯+*m*_*n*_ and *M* = *μ*_1_ + ⋯+*μ*_*n*_, *Re*(*s*) > 0 and *Re*(*υ* + *M*) > 0.

(b) Let $J_{2\mu_{j}}^{\left(2\right)}\left(2\sqrt{a_{j}t};q\right)$, *j* = 1,2,…, *n*, be *n* different *q*-Bessel functions and

(27) f(t)=tυ−1∏j=1nJ2μj(2)(2ajt;q).

Then, *q*-Sumudu transform of *f*(*t*) is

(28) Sq{f(t);s} =Γq(v+M)(1−q)v−M−1a1μ1⋯anμnsv+M−1Γq(2μ1+1)⋯Γq(2μn+1)  ×∑m1,…,mn=0∞(qv+M;q)m∏j=1nqmj(mj−1)(−q2μj+1ajs)mj(q2μ1+1;q)mj(q;q)mj,

where *m* = *m*_1_ + *m*_2_ + ⋯+*m*_*n*_ and *M* = *μ*_1_ + ⋯+*μ*_*n*_, *Re*(*s*) > 0 and *Re*(*υ* + *M*) > 0.

(c) Let $J_{2\mu_{j}}^{\left(3\right)}\left(\sqrt{q^{-1}a_{j}t};q\right)$, *j* = 1,2,…, *n*, be *n* different *q*-Bessel functions and

(29) $$\begin{array}{c} f\left(t\right)=t^{\upsilon -1}\prod_{j=1}^{n}q^{\mu_{j}}J_{2\mu_{j}}^{\left(3\right)}\left(\sqrt{q^{-1}a_{j}t};q\right). \end{array}$$

Then, *q*-Sumudu transform of *f*(*t*) is

(30) Sq{f(t);s} =(1−q)υ−M−1a1μ1⋯anμnΓq(υ+M)sυ+M−1Γq(2μ1+1)⋯Γq(2μn+1)  ×∑m1,…,mn=0∞(qυ+M;q)m∏j=1nqmj(mj−1)/2(−ajs)mj(q2μj+1;q)mj(q;q)mj,

where *m* = *m*_1_ + *m*_2_ + ⋯+*m*_*n*_ and *M* = *μ*_1_ + ⋯+*μ*_*n*_, *Re*(*s*) > 0 and *Re*(*υ* + *M*) > 0.

**Proof**

We will only give the proof of (26), because the proof of (28) and (30) is the same. We put

(31) $$\begin{array}{c} f\left(t\right)=t^{\upsilon -1}J_{2\mu_{1}}^{\left(1\right)}\left(2\sqrt{a_{1}t};q\right)\cdots J_{2\mu_{n}}^{\left(1\right)}\left(2\sqrt{a_{n}t};q\right) \end{array}$$

into the definition (17) and making use of (1) yields

(32) Sq{f(t);s} =(q;q)∞∑j=0∞qj(q;q)j(sqj)υ−1  ×{(q2μ1+1;q)∞(q;q)∞(a1sqj)μ1⋯(q2μn+1;q)∞(q;q)∞(ansqj)μn}  ×∑m1,…,mn=0∞(−a1sqj)m1(q2μ1+1;q)m1(q;q)m1  ×⋯(−ansqj)mn(q2μn+1;q)mn(q;q)mn.

On interchanging the order of summations, which is valid under the conditions given by the theorem, we obtain

(33) Sq{f(t);s} =(q;q)∞sυ+M−1∏j=1najμj(q2μj+1;q)∞(q;q)∞  ×∑m1,…,mn=0∞ ∏j=1n(−a1sqk)mj(q2μj+1;q)mj(q;q)mj∑j=0∞qj(υ+M+m)(q;q)j,

where *m* = *m*_1_ + *m*_2_ + ⋯+*m*_*n*_ and *M* = *μ*_1_ + ⋯+*μ*_*n*_. By using the property of (21) and then using the definition of *q*-exponential function, namely,

(34) (q;q)∞∑j=0∞qj(υ+M+m)(q;q)j=(q;q)∞(qυ+M+m;q)∞=(q;q)∞(qυ+M;q)∞(qυ+M;q)m,

we get the desired result. By applying the similar procedure as of Theorem 1(a), one can easily establish Theorem 1(b) and (c). Therefore, we omit the further details of the proof of this theorem.

**Theorem 2**

(a) Let $J_{2\mu_{j}}^{\left(2\right)}\left(2\sqrt{a_{j}t};q\right)$, *j* = 1,2,…, *n*, be n different *q*-Bessel functions and

(35) $$\begin{array}{c} f\left(t\right)=t^{\upsilon -1}\prod_{j=1}^{n}J_{2\mu_{j}}^{\left(2\right)}\left(2\sqrt{a_{j}t};q\right). \end{array}$$

Then, *q*-Sumudu transform of *f*(*t*) is

(36) 𝕊q{f(t);s} =Γq(υ+M)(1−q)υ−M−1Γq(2μ1+1)⋯Γq(2μn+1)a1μ1⋯anμnK(1/s;υ+M)sυ+M−1  ×∑m1,…,mn=0∞(qυ+M;q)m(qv+M)mqm(m−1)/2  ×∏k=1nqmk(mk−1)(−q2μk+1aks)mk(q2μk+1;q)mk(q;q)mk,

where *m* = *m*_1_ + *m*_2_ + ⋯+*m*_*n*_ and *M* = *μ*_1_ + ⋯+*μ*_*n*_, *Re*(*s*) > 0 and *Re*(*υ* + *M*) > 0.

(b) Let $J_{2\mu_{j}}^{\left(3\right)}\left(\sqrt{q^{-1}a_{j}t};q\right)$, *j* = 1,2,…, *n*, be *n* different *q*-Bessel functions and

(37) $$\begin{array}{c} f\left(t\right)=t^{\upsilon -1}\prod_{j=1}^{n}q^{\mu_{j}}J_{2\mu_{j}}^{\left(3\right)}\left(\sqrt{q^{-1}a_{j}t};q\right). \end{array}$$

Then, *q*-Sumudu transform of *f*(*t*) is given by

(38) 𝕊q{f(t);s} =Γq(υ+M)(1−q)υ+M−1Γq(2μ1+1)⋯Γq(2μn+1)a1μ1⋯anμnK(1/s;υ+M)sυ+M−1  ×∑m1,…,mn=0∞(qυ+M;q)m(qv+M)mqm(m−1)/2  ×∏k=1nqmk(mk−1)/2(−aks)mk(q2μk+1;q)mk(q;q)mk,

where *m* = *m*_1_ + *m*_2_ + ⋯+*m*_*n*_ and *M* = *μ*_1_ + ⋯+*μ*_*n*_, *Re*(*s*) > 0 and *Re*(*υ* + *M*) > 0.

**Proof**

We will only give the proof of (36), because the proof of (38) is the same. We put

(39) $$\begin{array}{c} f\left(t\right)=t^{\upsilon -1}J_{2\mu_{1}}^{\left(2\right)}\left(2\sqrt{a_{1}t};q\right)\cdots J_{2\mu_{n}}^{\left(2\right)}\left(2\sqrt{a_{n}t};q\right) \end{array}$$

into the definition (18) and make use of (36); then we have

(40) 𝕊q{f(t);s} =s−1(−(1/s);q)∞∑j∈ℤqj(−1s;q)j(qj)υ−1  ×{(q2μ1+1;q)∞(q;q)∞(a1qj)μ1⋯(q2μn+1;q)∞(q;q)∞(anqj)μn}  ×∑m1,…,mn=0∞‍qm1(m1−1)(−q2μ1+1a1qj)m1(q2μ1+1;q)m1(q;q)m1  ×⋯qmn(mn−1)(−q2μn+1anqj)mn(q2μn+1;q)mn(q;q)mn.

On interchanging the order of summations, which is valid under the conditions given by the theorem, we obtain

(41) 𝕊q{f(t);s} =s−1(−(1/s);q)∞(q2μ1+1;q)∞(q;q)∞⋯(q2μn+1;q)∞(q;q)∞a1μ1⋯anμn  ×∑m1,…,mn=0∞qm1(m1−1)(−q2μ1+1a1)m1(q2μ1+1;q)m1(q;q)m1  ×⋯qmn(mn−1)(−q2μn+1an)mn(q2μn+1;q)mn(q;q)mn  ×∑j∈ℤ(−s−1;q)jqj(υ+μ1+⋯+μn+m1+⋯+mn).

In the series representation of (24), if we write *A* = *s* and *α* = *υ* + *M* + *m*, where *M* = *μ*_1_ + ⋯+*μ*_*n*_ and *m* = *m*_1_ + ⋯+*m*_*n*_, we get

(42) 𝕊q{f(t);s} =(q2μ1+1;q)∞(q;q)∞⋯(q2μn+1;q)∞(q;q)∞a1μ1⋯anμn  ×∑m  1,…,mn=0∞qm1(m1−1)(−q2μ1+1a1)m1(q2μ1+1;q)m1(q;q)m1  ×⋯qmn(mn−1)(−q2μn+1an)mn(q2μn+1;q)mn(q;q)mn  ×(1−q)υ+M+m−1sυ+M+m−1K(s;υ+M+m)Γq(υ+M+m).

By using the properties of (21) and (22) and then using the following remarkable identity:

(43) $$\begin{array}{c} {\left(q^{t+m};q\right)}_{\infty }=\frac{{\left(q^{t};q\right)}_{\infty }}{{\left(q^{t};q\right)}_{m}}\;\left(m\in \mathbb{N}\right), \end{array}$$

we have

(44) 𝕊q{f(t);s} =Γq(υ+M)(1−q)υ−M−1Γq(2μ1+1)⋯Γq(2μn+1)a1μ1⋯anμnK(1/s;υ+M)sυ+M−1  ×∑m1,…,mn=0∞(qυ+M;q)m(qv+M)mqm(m−1)/2  ×∏k=1nqmk(mk−1)(−q2μk+1aks)mk(q2μk+1;q)mk(q;q)mk.

Again, by applying the similar procedure as of Theorem 2(a), one can easily establish Theorem 2(b). Therefore, we omit the further details of the proof of this theorem.

## 4. Illustrative Examples

In this section we evaluate the *q*-Sumudu transforms involving the *q*-Bessel functions as applications of our main results.

**Corollary 3**

If one takes *n* = 1, *μ*_1_ = *v*, *v* = *μ*, and *a*_1_ = *a* in above theorems, respectively, one has

(45) Sq{tμ−1J2v(1)(2at;q);s} =Γq(μ+υ)(1−q)μ−v−1Γq(2v+1)avsυ+μ−1  ×Φ21[qμ+υ0q2v+1;q,−as],Sq{tμ−1J2v(2)(2at;q);s} =Γq(μ+υ)(1−q)μ−v−1Γq(2v+1)avsυ+μ−1  ×Φ12[qμ+υq2v+10;q,−q2ν+1as],Sq{tμ−1qvJ2v(3)(q−1at;q);s} =Γq(μ+υ)(1−q)μ−v−1Γq(2v+1)avsυ+μ−1  ×Φ11[qμ+υq2v+1;q,as],𝕊q{tμ−1J2v(2)(2at;q);s} =Γq(μ+υ)(1−q)μ−v−1Γq(2v+1)K(s;μ+υ)avsυ+μ−1  ×Φ11[qμ+υq2v+1;q,q2v+1qμ+υas],𝕊q{tμ−1qvJ2v(3)(q−1at;q);s} =Γq(μ+υ)(1−q)μ−v−1Γq(2v+1)K(s;μ+υ)avsυ+μ−1  ×Φ21[qμ+υ0q2v+1;q,−asqμ+υ],

where *Re*(*s*) > 0 and *Re*(*μ* + *υ*) > 0.

**Corollary 4**

In Corollary 3, if one writes (*v*/2) + 1 and (*υ*/2) instead of *μ* and *υ*, respectively, one obtains

(46) Sq{tv/2Jv(1)(2at;q);s}=av/2sυeq(−as),Sq{tv/2Jv(2)(2at;q);s}=av/2sυΦ01[−0;q,−qν+1as],Sq{(qt)v/2Jv(3)(q−1at;q);s}=av/2sυEq(as),𝕊q{tv/2Jv(2)(2at;q);s}=av/2sυK(s;υ+1)Eq(as),𝕊q{(qt)v/2Jv(3)(q−1at;q);s}=av/2sυK(s;υ+1)eq(−asqυ+1),

where *Re*(*s*) > 0 and *Re*(*υ* + 1) > 0.

**Corollary 5**

In Corollary 4, if one chooses *v* = 1, respectively, one gets

(47) Sq{t1/2J1(1)(2at;q);s}=a1/2seq(−as),Sq{tv1/2J1(2)(2at;q);s}=a1/2sΦ01[−0;q,−q2as],Sq{(qt)1/2J1(3)(q−1at;q);s}=a1/2sEq(as),𝕊q{t1/2J1(2)(2at;q);s}=q−1a1/2s  Eq(as),𝕊q{(qt)1/2J1(3)(q−1at;q);s}=q−1a1/2seq(−q−2as),

where *Re*(*s*) > 0.

**Corollary 6**

Similarly, if one chooses *v* = 0 in Corollary 4, respectively, one gets

(48) Sq{J0(1)(2at;q);s}=eq(−as),Sq{J0(2)(2at;q);s}=Φ01[−0;q,−qas],Sq{J0(3)(q−1at;q);s}=Eq(as),𝕊q{J0(2)(2at;q);s}=Eq(as),𝕊q{J0(3)(q−1at;q);s}=eq(−q−1as),

where *Re*(*s*) > 0.

**Corollary 7**

In Corollary 3, if one writes *υ* = 0 and then *a* = 0, one finds

(49) Sq{tμ−1;s}=Γq(μ)(1−q)μ−1sμ−1,𝕊q{tμ−1;s}=Γq(μ)(1−q)μ−1K(s;μ)sμ−1,

where *Re*(*s*) > 0 and *Re*(*μ*) > 0.

**Remark 8**

The results in Corollary 7 are previously obtained in [9, page 418, (2.5)-(2.6)].

## 5. Concluding Remarks

We conclude this investigation by remarking that *q*-Sumudu transforms of many other *q*-Bessel functions can be evaluated in this manner by applying the above theorems and their various corollaries and consequences considered here. Also, the results obtained here will be used in the forthcoming paper where some *q*-difference equations are solved.
